# Bandwidth Constraints to Using Video and Other Rich Media in Behavior Change Websites

**DOI:** 10.2196/jmir.7.4.e49

**Published:** 2005-09-16

**Authors:** Brian G Danaher, Stephen A Jazdzewski, H Garth McKay, Clinton R Hudson

**Affiliations:** ^2^Oregon Center for Applied ScienceEugeneORUSA; ^1^Oregon Research InstituteEugeneORUSA

**Keywords:** Health behavior, Internet, behavioral research, rich media, video, smoking cessation, bandwidth usage index

## Abstract

**Background:**

Web-based behavior change interventions often include rich media (eg, video, audio, and large graphics). The rationale for using rich media includes the need to reach users who are not inclined or able to use text-based website content, encouragement of program engagement, and following the precedent set by news and sports websites.

**Objectives:**

We describe the development of a bandwidth usage index, which seeks to provide a practical method to gauge the extent to which websites can successfully be used within different Internet access scenarios (eg, dial-up and broadband).

**Methods:**

We conducted three studies to measure bandwidth consumption. In Study 1, we measured the bandwidth usage index for three video-rich websites (for smoking cessation, for caregivers, and for improving eldercare by family members). We then estimated the number of concurrent users that could be accommodated by each website under various Internet access scenarios. In Study 2, we sought to validate our estimated threshold number of concurrent users by testing the video-rich smoking cessation website with different numbers of concurrent users. In Study 3, we calculated the bandwidth usage index and threshold number of concurrent users for three versions of the smoking cessation website: the video-rich version (tested in Study 1), an audio-rich version, and a Web-enabled CD-ROM version in which all media-rich content was placed on a CD-ROM on the client computer.

**Results:**

In Study 1, we found that the bandwidth usage index of the video-rich websites ranged from 144 Kbps to 93 Kbps. These results indicated that dial-up modem users would not achieve a “good user experience” with any of the three rich media websites. Results for Study 2 confirmed that usability was compromised when the estimated threshold number of concurrent users was exceeded. Results for Study 3 indicated that changing a website from video- to audio-rich content reduced the bandwidth requirement by almost 50%, but it remained too large to allow satisfactory use in dial-up modem scenarios. The Web-enabled CD-ROM reduced bandwidth requirements such that even a dial-up modem user could have a good user experience with the rich media content.

**Conclusions:**

We conclude that the bandwidth usage index represents a practical tool that can help developers and researchers to measure the bandwidth requirements of their websites as well as to evaluate the feasibility of certain website designs in terms of specific use cases. These findings are discussed in terms of reaching different groups of users as well accommodating the intended number of concurrent users. We also discuss the promising option of using Web-enabled CD-ROMs to deliver rich media content to users with dial-up Internet access. We introduce a number of researchable themes for improving our ability to develop Web-based behavior change interventions that can better deliver what they promise.

## Introduction

The Internet holds great promise as a delivery channel for programs designed to help people change their behaviors [1,2]. For example, a number of reports have described encouraging results for Web-based interventions for quitting smoking [3-7], managing diabetes [8], managing depression and stress [9,10], and increasing exercise and losing weight [11,12]. An early review of this burgeoning field concluded that Web-based interventions were relatively more helpful than non-Web controls across a broad range of behaviors and methodologies [13]. One of the more impressive features of Web-based behavior change programs is their ability to incorporate rich media components that use video and audio.

In this report we will review some of the forces that encourage the development of rich media websites, but we will also examine in considerable detail the factors that temper unbridled enthusiasm for this trend. We believe that any reasoned analysis of the use of rich media website content should also consider the possible barriers to its use. As a key part of our analysis, we will describe the development and testing of a practical index for gauging the amount of bandwidth that a Web-based program requires. We hope to highlight the ways that bandwidth constraints associated with rich media restrict both program reach as well as the number of concurrent end users who can successfully use a Web-based program. We also discuss ways that behavioral research can help elucidate how and when to use different website program ingredients, including, for example, the use of rich media. The remainder of this section describes the trends encouraging the use of rich media and introduces key technology issues that set the stage for the presentation of our investigation.

### Trends and Assumptions That Encourage Use of Rich Media

**Figure 1:**
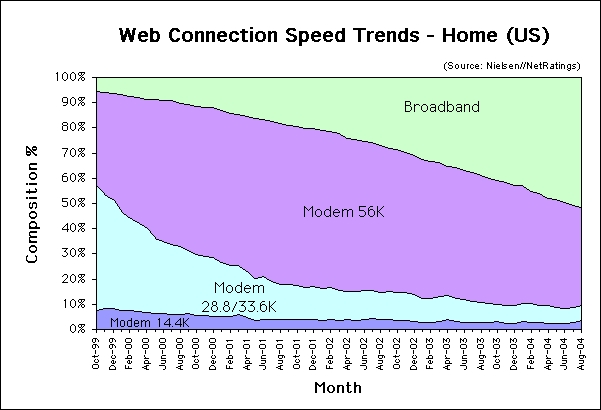
Web connection speed trends in US homes (used with permission [21])

Developing rich media is an expensive proposition [14]. Nonetheless, the use of rich media in Web-based programs has been encouraged by complementary technology trends: (1) the dramatic reduction in the cost and complexity of rich media recording and editing tools, (2) the fact that rich media is a defining characteristic of CD-ROM multimedia training [15-17], (3) the emergence of programming tools that reduce the complexity of delivering audio and video in websites, (4) the use of rich media content in a number of commercial websites (eg, sports, news, and entertainment sites) and company intranets [18], and (5) the growing number of households with *broadband* Internet access both in the United States [19] and the world [20]. Currently, more than 204 million people in the United States have home Internet access [19]. In August 2004, 51.4% of active Internet users connected from home using broadband connections. Among *narrowband* users, 39% use 56 Kbps modems, 6% use 28/33.3 Kbps modems, and 3.6% use 14.4 Kbps modems [19,21]. In summary, just under half of home users in the United States connect to the Internet at 56 Kbps or less (Figure 1).

The emergence of rich media websites has also been encouraged by a number of assumptions made by behavior change program developers: (1) online video and audio presentations help reach the audience that is not able or inclined to interact with websites that use text-based content; (2) rich media may be more effective than text in conveying emotional content and subtle interpersonal communications [22]—issues that may be particularly pertinent to many behavior change programs; (3) by varying rich media components, Web-based programs can deliver tailored content that is more closely aligned with the racial, ethnic, and demographic characteristics of users (eg, varying the ethnicity or age of video models can affect acceptability and impact [23]); and (4) the entertainment value of rich media can help overcome problems of participant recruitment and program engagement that seem to afflict many Web-based programs. A recent study on cancer communication materials found that participants preferred multimedia which combined text, spoken audio, and animation compared with presentations that involved text only, audio only, or text and synchronized audio [24,25].

### Technical Considerations

In this section we briefly introduce a number of technical considerations that affect website bandwidth constraints, including the size of rich media, ways to deliver rich media, calculating the extent of concurrent website usage, and typical bandwidth bottlenecks. Our discussion will only briefly touch upon the vast wealth of technological detail and resources associated with Internet content delivery/distribution. We encourage interested readers to explore this realm by first consulting the useful framework presented by Bush and his colleagues [26], followed by review of several excellent texts [27-29].

### Web-Delivered Rich Media

Rich media content delivered over the Web typically undergoes a number of changes in order to reduce its absolute size (number of bits of data) while maintaining an acceptable level of clarity and attractiveness (fidelity to the original) [30-32]. The smaller the data size of a media file, the more rapidly it can be delivered over the Internet to the end user. One approach to reducing the data size of rich media involves scaling, that is, reducing the frame or image size, the frame rate, or the color resolution [27]. In addition to scaling, data size can be reduced by using very sophisticated technologies that compress (remove information that is perceptually redundant [27]) media content with varying degrees of fidelity and acceptability [28]. Video can be delivered using a *progressive download* process or via *streaming*. In our experience, progressive download provides better fidelity with fewer interruptions and fewer problems synchronizing video with audible speech (correctly timing speaker’s lips and voice). Synchronization problems of this type are very distracting, and, as a result, they significantly degrade usability [33]. Finally, it is possible for websites to *preload* or *push* rich media to the end user before such content is selected for viewing [34], but this approach works best when the user has a broadband connection and when rich media selections are few in number or can be predicted reliably ahead of user selection (not typical of rich media behavior change websites). Corporate examples of preloading can be found in media content stored in cache on proxy servers [27].

### Concurrent Users

Website usability is greatly affected by the number of users who access content at the same time. As a general rule, because of the asynchronous use pattern associated with most Web-based programs, the number of concurrent users is a small percentage of the total number of users eligible to access the program. However, given that websites can be made available to and thus accessed by an extremely large number of users, even a small percentage can yield a relatively large absolute number of concurrent users. As Salchner [35] has noted, concurrent user estimates can be derived by calculating the average number of user visits that occur each day and the average number of users per hour that a program can support (60 divided by the duration [in minutes] of a typical session or visit). Of course, visits are not evenly distributed across the hours of the day. And each user does not access the same program content (especially in Web-based programs with matrix information architecture components that enable users to choose how they access program content [2]). In summary, the number of concurrent users is critically important to website usability since it interacts with the available bandwidth (described next).

### Bandwidth Bottlenecks

Most users and program developers are familiar with the term *bandwidth*, which is defined as “the amount of data that can be transferred through a digital connection in a given time period…[which is] usually measured in bits or bytes per second” [36]. The physical metaphor of a pipe is often invoked in an effort to describe the manner in which digital connections behave: larger pipes have greater bandwidth and they permit faster throughput of the digital data. As with the limits imposed by physical pipes, there are limitations on the amount of data that can be transmitted through any digital connection.

Bandwidth constraints have significant practical implications because, when exceeded, they can greatly increase end user frustration and seriously degrade interaction with a website. Consider, for example, that usability research confirms that users have extremely limited patience when waiting for a Web page to download [37]. The longer the wait, the less acceptable—and hence the less usable—the website. Jakob Nielsen, a noted Web usability expert, has recommended that response times of less than 0.1 second are needed for the user to feel that the system is being responsive (that the system has “heard” and “responded” to the user’s request) and that any delay of greater than 1.0 second will interrupt the user’s flow of thought [38].

**Figure 2:**
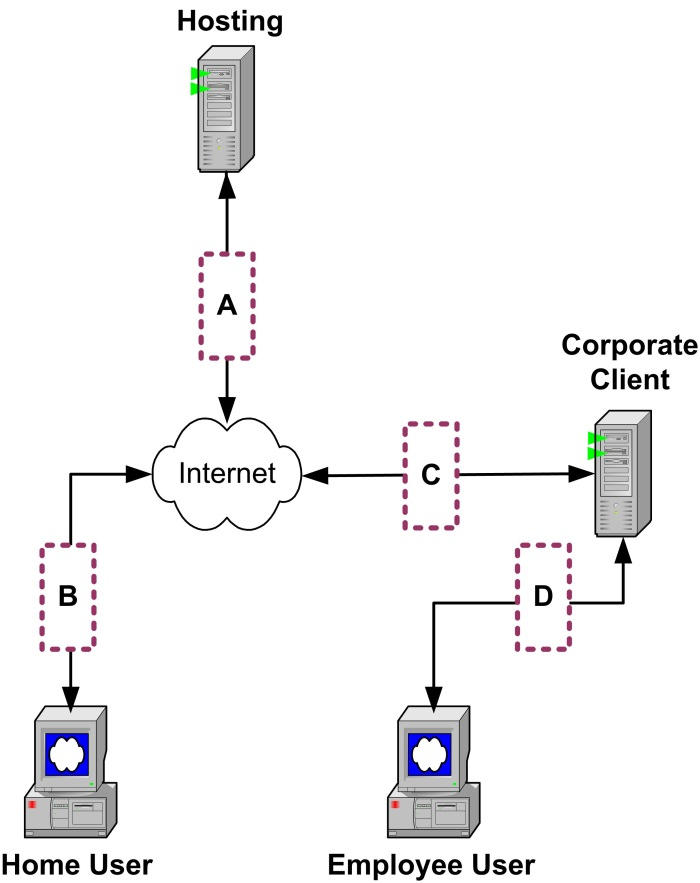
Potential bandwidth bottlenecks

There are at least four potential delivery bottlenecks that can interact in a cumulative fashion to impede the acceptable flow of content from the Internet to the end user [39]. The size/speed of the digital connection at various key points in the content delivery process has a very significant impact on bandwidth. Figure 2 depicts the key points where throughput bottlenecks can occur.

Bottleneck A is defined as the capacity of the host to serve (deliver) the program content. Throughput can be undermined at this point if the program content is being served by an inefficient or overworked server. Fortunately, current servers are sufficiently robust that they become a throughput bottleneck only in scenarios when they are called upon to manage responses and generate logic to build and delivery dynamic Web pages for a very large number of concurrent users. Moreover, there are sophisticated hardware and software solutions for handling this type of demand. For example, major commercial Internet content providers typically use a server farm configuration in which one group of servers manages applications (program logic), other servers process database activities, and yet other servers manage the delivery of media assets (eg, graphics, audio, and video) [40]. However, when many concurrent users access video-rich content, the bottleneck is defined by the bandwidth constraints that cannot be resolved by improvements in server hardware configuration.

Bottleneck B describes the connection between the Internet and the home user. It is possible that the user’s computer can be underpowered to manage Web-based programs with rich media. But a critically important factor at this point involves the “last mile” challenge [41,42] (ie, bandwidth limitations in delivering Internet content from the service provider to the home user). The limited bandwidth of dial-up modem users represents an obvious example of this bottleneck. So-called *broadband* connections enable home users to download significantly greater amounts of data per unit time from the Internet.

Bottleneck C describes the connection between the Internet and a company network. Many companies use T1 lines that provide all employees with “high speed” access to the Internet, email, and data sharing.

Bottleneck D describes the connection between a company network and the individual employee workstation.

### Summary

Rich media is becoming a frequent ingredient of Web-based behavior change programs. As we have noted, there are forces that encourage this trend, as well as associated bandwidth requirements that need to be measured in order to inform the optimal planning, development, and delivery of Web-based programs that use rich media.

## Methods

### Study 1: Three Different Web Programs

We used a relatively simple methodology to calculate the bandwidth requirements of an Internet-based program. The resulting metric, which we have labeled the bandwidth usage index (BUI), can be used to define the minimum bandwidth requirements for any given program and can be used to estimate the number of concurrent users that can be accommodated (have a good user experience) under varying Internet access scenarios (eg, ranging from using a dial-up modem to using an internal network).

**Figure 3:**
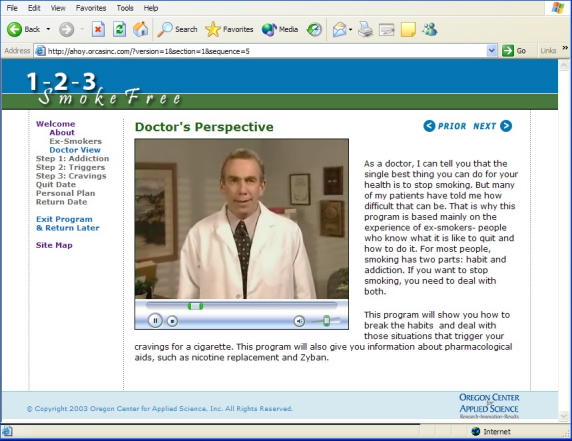
1-2-3 SmokeFree Web page showing video and text components

We determined the BUI for three different video-rich Web-based behavior change programs: (1) the 1-2-3 SmokeFree smoking cessation program [43] (Figure 3), (2) a program designed for caregivers, and (3) a program designed to improve eldercare by family members. Each of these programs was developed with NIH Small Business Innovation Research funding at the Oregon Center for Applied Science, each used a modularized, data-driven design that displayed content based upon the interaction of logic scripts (eg, PHP), SQL databases, and cascading style sheets, and each made extensive use of video and audio (rich media) components. In all tests, video was delivered using the progressive download method via a Windows Media Player object embedded in the Web page.

Specifically, we measured the amount of data received by an end user (client computer) from a server (host computer) that delivered representative content from each of the three rich media Web-based programs. The 13-minute test followed a consistent protocol described generically in Table 1 and in more technical detail (with an example) in Multimedia Appendix 1. All tests were conducted in a 100 Mbps Ethernet network, which essentially eliminated any meaningful artifacts that would be associated with delays in network transmission (server response and bandwidth constraints).

**Table 1:** BUI measurement protocol

**Step**	**BUI Measurement Task**
1.	Clear the browser cache (from MS Internet Explorer 5.x, select “Tools,” Internet Options,” “General” tab, and click on “Delete Files” button).
2.	Access statistics for the network port that will be used by the test computer.
3.	Determine the noise level during a period of inactivity (see Multimedia Appendix 1).
4.	Reset the byte counters or settings for the port connected to the test computer.
5.	Launch and then use the Web-based behavior change program in a representative manner for the designated test period.
6.	Calculate the bytes received by the test computer at the end of the test period.
7.	Subtract the predicted noise from the bytes received calculation.
8.	Convert (as needed) into Kbps to obtain the BUI.

It is important to note that the end user in this test accessed a representative portion of the behavior change program content hosted on the server. We defined “representative portion” as a mixture of Web pages which included text, graphics, and rich media content (audio and video) used in a manner that simulated a typical use case. The index or gauge of data throughput was calculated as the total number of bits received by the client computer from the server divided by the duration of the test period to yield an average expressed in Kbps.

We used the BUI calculations to predict the number of concurrent users who could have a satisfactory experience using Web-based behavior change programs under a number of different Internet access scenarios.

### Study 2: BUI Validation Tests

In Study 2, we attempted to confirm the validity of our estimated threshold number of concurrent users for the three rich media behavior change websites we tested in Study 1. First, we used operating system tools to *throttle* a network computer (host) so that it would allow only 768 Kbps throughput (equivalent to the maximum throughput that would be experienced when using a DSL/cable to access the Internet). Next, we used that network computer to host the video-rich 1-2-3 SmokeFree Web-based smoking cessation program tested in Study 1. We stationed each of five end user participants (blind as to our earlier calculations and hypotheses) in separate offices where they could receive study instructions. Each end user participant used a separate workstation connected to the company intranet, and each was asked to clear the browser cache (Step 1 in Table 1). Finally, each participant was instructed at the same time via the intercom to launch and then use the Web-based behavior change program in a representative manner, which was described to them in terms of activities to perform and pages to visit (Step 5 in Table 1). At the end of the 5-minute test period, each participant was asked to rate the quality of their experience with the Web-based program using the binary label of “a good experience”—the program pages flow with little or no delay—or a “bad experience.”

### Study 3: Varying BUI in Three Versions of the Same Web Program

We also tested the extent to which the bandwidth requirements of a single Web-based program might vary based upon the type of rich media it presents and its delivery format. More specifically, we created two new versions of the 1-2-3 SmokeFree Web-based smoking cessation program: (1) an “audio-rich” version in which program content had been adapted such that video content was replaced by audio-only content, and (2) a Web-enabled CD-ROM condition in which all media-rich content was placed on a CD-ROM that was located on the client computer. All program content and behavior was otherwise identical to that found in the video-rich version of the smoking cessation program tested in Study 1. We followed the test protocol described in Table 1 to obtain throughput measures (the BUI) for each of these new versions in order to determine how they compared to the video-rich version.

## Results

### Study 1: Three Different Web Programs

The BUI for three video-rich Web-based behavior change programs ranged from 144 Kbps for the smoking cessation website to 93 Kbps for the eldercare website (Table 2). By dividing the total amount of data that can be transmitted (based upon the type of Internet connection) by the BUI (the amount of data per second being transmitted by the program), we were able to estimate the number of concurrent users who could use the Web-based program content. It is important to note that we used the *idealized* throughput of various methods of accessing the Internet, ranging from the slowest 28.8 Kbps dial-up modem to an extremely fast 100-baseT corporate network [44,45], to calculate the estimated number of concurrent users who could be accommodated for each website. Many factors affect throughput availability (eg, network noise, competition from other applications or other users, and server and end user hardware and system configurations). Actual throughput is almost always less (never greater) than these idealized numbers. As a result, a more precise calculation of the maximum number of concurrent users would require the BUI (the focus of this report) and a representative measure of throughput availability.

In terms of Bottleneck A (Figure 2), Table 2 provides a useful guide for how many concurrent users an Internet host could accommodate. Internet service providers (ISPs) typically offer a range of services, including increasing levels of bandwidth at correspondingly higher costs. If the ISP offers Internet access equivalent to T1 bandwidth, then Table 2 shows that the ISP could host only 10 to 16 concurrent users of the rich media programs (eg, the smoking cessation program had 1544 Kbps/144 Kbps/user = 10 users [an integer]).

In terms of Bottleneck B describing end user access to the Internet, data displayed in Table 2 show that users with a dial-up modem (either 28.8 or 29 Kbps or 56 Kbps) would not be able to achieve a “good user experience” with any of the three rich media website programs. In order to use any of these programs, the end user would need to have at least a DSL/cable broadband connection.

In terms of Bottleneck C, Table 2 indicates that all Internet activity would be brought to a halt in a company with a single T1 access to the Internet if 17 or more of its employees were to concurrently access any of the rich media websites. Moreover, the same result would be obtained if only 11 employees were to concurrently access the smoking cessation program. Generally speaking, companies are loath to have mission-critical business activity interrupted in this manner.

**Table 2:** Estimated number of concurrent users accommodated by three behavior change Web-based programs, by type of Internet connection

**Type of Internet Access and Related Maximum Download Throughput**		**Concurrent Users of Rich Media Web-Based Programs**		
		**Smoking cessation**	**Caregiver**	**Eldercare**
**Connection Type**	**Kbps**	**BUI = 144 Kbps**	**BUI = 95 Kbps**	**BUI = 93 Kbps**
Dial-up	29	0	0	0
Dial-up	56	0	0	0
DSL/cable	384	2	4	4
DSL/cable	768	5	8	8
T1	1544	10	16	16
Home WIFI from cable modem	1972	13	20	21
Home cable modem	3000	20	31	32
T3	44736	310	470	481
Internal network (100-baseT)	100000	694	1052	1075

Finally, considering Bottleneck D, data presented in Table 2 underscore the fact that the remarkable speed of network connections means that this connection would rarely become a bandwidth bottleneck. This conclusion would seem to recommend that rich media programs should be placed on a company server or, alternatively, on a server that is connected inside the company firewall. However, few corporate IT departments wax enthusiastic about outside servers being connected to their networks, and there is also the challenge of outside organizations being able to maintain and update these servers within idiosyncratic IT environments. It is also important to note that Web-based programs are typically developed and then demonstrated using corporate networks that present few bandwidth constraints. Tests on corporate networks can lull the user/client/developer into the mistaken conclusion that the program will give a good user experience when it is delivered over the Internet.

### Study 2: BUI Validation Tests

When 5 participants accessed the video-rich version of the smoking cessation program using a maximum download bandwidth of 768 Kbps, all users reported that they had a good experience with the program. The situation changed dramatically when 6 participants concurrently accessed the same program from within the same network environment—in this test case, all users reported that they had an unsatisfactory experience using the website, with reports of long delays in page loading, videos not playing, and so forth. These results confirmed the validity of the threshold numbers described in Table 2. Specifically, using the metrics in Table 2, it is possible to predict the number of concurrent users that will have a satisfactory experience. Exceeding that threshold number completely retrogrades the satisfactory experience for each concurrent user. The effect is not partial but, rather, it leads to a complete inability to access the program.

### Study 3: Varying BUI in Three Versions of the Same Web Program

Results of the third study are presented in Table 3. Note that changing a program from video to audio reduced bandwidth requirements by almost 50% (from a BUI of 144 Kbps to 65.9 Kbps), but this bandwidth requirement was still too large to enable an end user to access this audio-rich Web-based content using a dial-up modem. In marked contrast, using the Web-enabled CD-ROM reduced bandwidth requirements dramatically (from a BUI of 144 Kbps to 2.1 Kbps), which would enable a user to easily access rich media content even from a dial-up modem. It is also important to note that even if a Web-enabled CD-ROM were to be used, the hardware and software constraints become increasingly salient when Internet access speeds increase (eg, T3 and faster), thereby enabling more concurrent users to access hosted content. Consider, for example, that Table 3 indicates that by using a T3 access to the Internet and a Web-enabled CD-ROM program format it might be possible to host more than 21000 concurrent users. In such circumstances, greater attention must be afforded to the number and type of server configurations (hardware and software) needed to generate logic, select content, and build dynamic Web pages.

**Table 3:** Estimated number of concurrent users accommodated by three variants of the same Web-based smoking cessation program, by type of Internet connection

**Type of Internet Access and Related Maximum Download Throughput**		**Concurrent Users of Variants of a Web-Based Smoking Cessation Program**		
		**Video-Rich**	**Audio-Rich**	**Web-Enabled CD-ROM**
**Connection Type**	**Kbps**	**BUI = 144 Kbps**	**BUI = 65.9 Kbps**	**BUI = 2.1 Kbps**
Dial-up	29	0	0	13
Dial-up	56	0	0	26
DSL/cable	384	2	5	182
DSL/cable	768	5	11	365
T1	1544	10	23	735
Home WIFI from cable modem	1972	13	29	939
Home cable modem*	3000	20	45	1428
T3	44736	311	678	21302
Internal network (100-baseT)	100000	694	1517	47619

^*^ Faster cable modem download speeds up to three times this figure are emerging as providers ease bandwidth restrictions.

## Discussion

In this report we have described a series of tests of the bandwidth usage index (BUI) that confirm the value of measuring the actual data throughput associated with delivering Web-based behavior change programs that have varying amounts of rich media. We believe that the BUI or other similar throughput measures can provide designers with important guidance about how much rich media to include in their Web-based programs depending upon the intended audience and the intended number of concurrent users.

### Diffusion of Technology Changes

While technology changes at a very rapid rate, it is nonetheless also true that program designers need to take into careful consideration the significant delays associated with the diffusion of these changes to the large installed base of computer users. This challenge is confronted when considering end user software and hardware (eg, types and versions of operating systems, browsers, and browser plug-ins like Macromedia Flash and Adobe Acrobat; typical monitor sizes; and screen resolution settings). It is also highly relevant when considering types of Internet access which, in turn, affect the choice of program ingredients. These considerations may be even more salient when a Web-based intervention is targeted to groups that are not as far ahead as others on the adoption curve—as in the limited broadband penetration in rural [46] and different ethnic and racial communities [47]. Moreover, the adoption rate for broadband Internet access may slow down [21,48].

### Tradeoffs to Delivering Digital Content

Delivering digital content is fraught with tradeoffs regarding costs, reach, and scalability. If a Web-based program uses rich media content, then the calculations we describe in this report indicate that the program will not be usable for approximately half of US Internet users who still use dial-up modems. Even if you target a website to users whose broadband Internet access provides them with more than sufficient bandwidth to receive rich media programming, the hosting of that rich media content to thousands of concurrent users has substantial costs associated with using content delivery network solutions (eg, Akamai [49] and Cisco Systems [40]). Commercial websites subsidize such outsourcing by selling paid Web page advertisements—a business model that may not be feasible or even appropriate for many behavior change websites.

### Recommendations for Future Research

We recommend that reports of Web-based programs should routinely provide an indicator of bandwidth consumption, like the BUI, in order for readers to evaluate the manner in which such programs can be used. Of course, not all Web-based programs need to be designed for large audiences. But those programs that have widespread use as their goal need to be designed—and then fully tested—in a manner that ensures they can deliver the goods consistent with their intentions.

As we noted in our introduction, we believe that empirical research needs to examine the accuracy of widely held assumptions that video and audio automatically add value to Web-based programs [50,51]. Consider, for example, that intriguing research by Reeves and Nass [33] suggests that the value of the verisimilitude of video presentations may be overstated. Their studies suggest that video is not as critical an ingredient in the design of computer-based programs as is the tone of the communication that can be tailored to fit the user’s expectations. Fogg [52] echoes this point in his discussion of the persuasive features of the computer as *social actor*. It is reasonable to expect that many users would not be well served by websites in which they become passive page turners while observing multiple “talking head” presentations [53]. Similarly, behavioral researchers have a very significant opportunity to explore and test how best to use the interactivity and tailoring possibilities of Web-based program delivery (eg, [54]).

Future research should consider developing *lean* *media* websites that use vector-based animated graphics to offer interactivity and graphical content without excessive bandwidth consumption. One vector graphics tool to consider in this regard is Macromedia Flash (for its vector graphics capabilities rather than its video capabilities). Flash uses a client-side browser plug-in that has a very large installed base: version 2.0 is on 98.3% of current computers, whereas the most recent version 7.0 is on 90.0% of current computers [55]. Alternatively, it is possible to use an open source structured vector graphics (SVG) tool rather than the proprietary Flash format, but currently only 13% of users have the SVG browser plug-in on their computers [55]. The acceptability of websites may be compromised when users are required to download and install new or updated versions of browser plug-ins, especially in scenarios in which the download is large and users have limited bandwidth, dial-up Internet access.

Researchers and developers of Web-based behavior change programs should consider using intelligent adaptive designs that “sniff out” user’s bandwidth transparently and then tailor program content according to the bandwidth characteristics of each user’s Internet connection. Using this approach would require program designers to consider how best to deliver equivalent content using different media—a topic with both technical and behavior change complexities that have received little empirical study to date.

Website designers should make greater use of Web-enabled CD-ROMs to deliver rich media to the very large base of Internet users who do not have broadband access. Programs of this type would still use centralized functions of a Web-based program (eg, logic, authentication, and data collection), but they would access rich media located on each user’s computer. In this scenario it should be noted that video and audio would not need to be as compressed as for Web delivery. As a result, rich media components could be longer and larger (eg, larger video image), and their enhanced quality could result in better acceptability and improved impact.

Plans to market a Web-based program need to consider the bandwidth demands that might accompany program recruitment announcements or delivery scenarios that encourage concurrent usage. Consider, for example, the possible stress on a smoking cessation website tied to an event that occurs on a single date (eg, the Great American Smokeout) or when an announcement is issued to all employees of a large corporate client. Similarly, consider the scenario in which scores of users are assembled in community computer centers or corporate training rooms to access rich media Web-based programs. Announcements via mass media, email as well as URL publishing, can generate large demand spikes. Resulting peaks of Internet traffic, especially when programs deliver rich media content, can compromise the functioning of any Web-based program.

### Limitations and Strengths of Current Study

There are some noteworthy limitations to the design of the current study. The tests that validated the BUI threshold (number of concurrent users who would have a satisfactory user experience) were not exhaustive. More users could have been included to provide further confidence in the results we report. Nor was it possible for us to qualify the use of BUI by quantifying the peaks and valleys of available bandwidth in different real-world circumstances (eg, phone line degradation [particularly germane of dial-up and DSL users], geographically distributed users, times of day, server and end user hardware/software configurations). It is also important to acknowledge the subjective nature of the “satisfactory user experience” criterion we used. This measure was based upon individual user reactions when accessing program content in a realistic manner. We attempted to operationally define our use cases to be similar to what would typically be experienced by users of these programs.

Our investigation does, however, have a number of strengths. For example, we were able to measure the bandwidth use of a number of Web-based behavior change programs that contained rich media. In addition, we were able to test the impact on bandwidth use of three variations of one of these rich media behavior change programs, which enabled us to recommend the approach of using Web-enabled CD-ROM design to extend the reach of rich media Web programs to the largest possible audience of potential users. Finally, while the BUI is not a precise tool, it nonetheless describes a process that others can replicate in order to evaluate their own Web-based programs. We believe that important implications can be drawn from using the BUI to calculate estimates of the maximum number of concurrent users by Internet access type (Tables 2 and 3).

### The Scalability of Behavior Change Websites

Because they attract relatively meager numbers of concurrent users, typical randomized controlled trials of Web-based programs provide an inadequate test of their ability to deliver content in a scalable manner. In some ways, it is as if the airworthiness of a modern airliner is tested with a light load of passengers and fuel in only ideal flying conditions without ever being tested with a full load in windy, stormy conditions. But website scalability is a typical rationale invoked in support of using the Internet as a delivery channel and in justifying the considerable development expenses. We believe it is essential to acknowledge that Web-based programs need to be designed to fit the goals for which they are intended and that their bandwidth requirements need to be considered, measured, and reported in order to gauge how well they will likely operate under their intended use case scenarios.

## Multimedia Appendix 1

Calculation of BUI.

## Abbreviations

BUI: bandwidth usage index
Kbps: kilobits per second
NIH: National Institutes of Health
PHP: PHP hypertext preprocessor
