# Supplementary material for: Bandwidth Constraints to Using Video and Other Rich Media in Behavior Change Websites
Source: J Med Internet Res. 2005 Sep 16;7(4):e49. doi: 10.2196/jmir.7.4.e49 (PMC1550668; doi:10.2196/jmir.7.4.e49)
Supplement: Supplementary file 1 [file jmir_v7i4e49_app1.pdf]

Calculation of BUI

The bandwidth usage index (BUI) is based upon a protocol that measures the number of bits of data received by the end user when accessing a Web-based program, in the following manner: (1) accesses a representative mixture of program content including text, graphics, and rich media; (2) produces a "good experience" as measured by the user being able to access intended content within a satisfactory response time; and (3) occurs over a designated test period.

As might be expected in measuring network activity, there are many technology tools and approaches that one might use to measure throughput and, consequently, BUI. We used a HP ProCurve Switch 4104gl Ethernet switch with software counters that could be set (and reset) to measure the number of bits received from the hosting computer by the test computer. In addition, we used these counters to measure the noise or protocol overhead that resulted in measurable bit traffic being received by the test computer that was not attributable specifically to the hosting of the Web-based program. This noise, or overhead, is important to acknowledge since it serves as a reminder that, in real world scenarios, BUI would compete with other network applications and activity for a finite amount of bandwidth. We calculated noise and protocol overhead by subtracting such data from the bits actually received in order to derive the bits received attributable to the Web-based program of interest. A more detailed description of the steps we used for measuring BUI is provided below.

It is important to note that BUI measurements should occur within a network environment that provides minimal delays in network transmission in order to make the throughput measurement accurate and representative. All tests we describe in this report were conducted in a 100 Mbps Ethernet network, which essentially eliminated any artifacts that would be associated with delays associated with network transmission.

Step 1: Open a terminal connection to the Ethernet switch and reset the counters for the port connected to the test computer.

Step 2: Measure noise and network protocol activity by counter settings recorded after no computer activity for 10 minutes.

Step 3: Clear cache as specified in Table 1.

Step 4: Reset port counters again to commence test period.

Step 5: Record port counters again at the end of the test period. This provides the total sent and received data in bytes during the test period. This is the actual data bytes received with protocol overhead subtracted.

Step 6: Reduce the number obtained in Step 5 by the observed noise level (numerical result of Step 2) to yield the final count of actual bytes received due to program use.

Step 7: This total is then expressed as bits (numerical result of Step 6 times 8 [bits per byte]).

Step 8: This total is then expressed as bits per second, or bps (numerical result of Step 7 divided by seconds in test period).

Step 9: This total is then converted to Kbps (numerical result of Step 7 divided by 1000) to yield BUI. (Filipov [44] notes that "some sources define a kilobit to mean 1024 bits. Although the bit is a unit of the binary number system, bits in data communications are discrete signal pulses and have historically been counted using the decimal number system.")

The content below shows log data from the test of the Web-Enabled CD-ROM program described in Table 3.

```
stevej@user ~ sudo /usr/bin/minicom
HP ProCurve Switch 4104GL# clear statistics  b2
HP ProCurve Switch 4104GL# show interfaces  b2
   Bytes Rx        : 678                    Bytes Tx        : 15206
   Unicast Rx      : 8                      Unicast Tx      : 15
   Bcast/Mcast Rx  : 0                      Bcast/Mcast Tx  : 126
   FCS Rx          : 0                      Drops Rx        : 0
   Alignment Rx    : 0                      Collisions Tx   : 0
   Runts Rx        : 0                      Late Colln Tx   : 0
   Giants Rx       : 0                      Excessive Colln : 0
   Total Rx Errors : 0                      Deferred Tx     : 0

HP ProCurve Switch 4104GL# clear statistics  b2
HP ProCurve Switch 4104GL# show interfaces  b2
   Bytes Rx        : 128510                 Bytes Tx        : 406662
   Unicast Rx      : 549                    Unicast Tx      : 699
   Bcast/Mcast Rx  : 7                      Bcast/Mcast Tx  : 1328
   FCS Rx          : 0                      Drops Rx        : 0
   Alignment Rx    : 0                      Collisions Tx   : 3
   Runts Rx        : 0                      Late Colln Tx   : 0
   Giants Rx       : 0                      Excessive Colln : 0
   Total Rx Errors : 0                      Deferred Tx     : 1
```

Step 2: Noise measured to be 15206 * 13 minutes = 197678

Step 5: Bytes received by the client computer (Bytes Tx from the switch) = 406662 bytes

Step 6: Approximate number of bytes due to program use = 406662 – 197678 = 208984 bytes

Step 7: Total bits = 208984 * 8 = 1671872 bits

Step 8: Total bits/second = 1671872 / 780 [second in test period] = 2143 bps

Step 9: BUI during a "good experience" is 2143 / 1000 [bits in Kbps] = 2.14 Kbps
